# Perceptions of community safety and social activity participation among youth in South Africa

**DOI:** 10.1371/journal.pone.0197549

**Published:** 2018-05-18

**Authors:** Nicole De Wet, Oluwaseyi Somefun, Ndivhuwo Rambau

**Affiliations:** Demography and Population Studies, University of the Witwatersrand, Johannesburg, South Africa; Public Library of Science, UNITED KINGDOM

## Abstract

**Background:**

Crime and violence causes massive disruptions to the health, survival and development of populations. In South Africa, incredibly high rates of crime and violence are noted. The country also has a very large youth population whose health, survival and development are key to economic growth. Among other efforts to encourage healthy youth development and the promotion of social activities such as sports, youth groups, choirs and so forth. This study examines the relationship between perceived community safety and the uptake of social activities among youth in South Africa.

**Data and methods:**

This paper uses data from the National Youth Lifestyle Survey (2008) with an unweighted sample of 4,391 youth (age12-22 years old). Using chi-square and logistic regression analysis the association between perceived community safety and social activity participation are tested.

**Findings:**

The results indicate that youth participation in social activities in South Africa is high (55% of males and 45% of females). Among males, the most prominent activity is sports (51.8%), while for females there is high participation in choir and singing groups (55.68%). More than 50% of males perceive their communities as risky while less than half of females feel the same. Male youth are more likely to participate in social activities if they perceive their communities as risky (OR = 1.04). Females (OR = 0.83), youth have a negative view of their future (OR = 0.43) are less likely to participate in social activities.

**Conclusions:**

There exists an association between youth’s perception of community safety and their participation in social activities. Whether sports and groups are protective or enabling environments for youth from unsafe communities is moot. More in-depth research is needed on why youth participate in these clubs and groups to truly understand the role of social activities in South African societies.

## Introduction

Crime and violence in South Africa has been noted as a serious problem. More recent statistics show 2,250,257 crimes reported for 2015 alone [1]. Further all crimes have increased since 2013, when 2,217,862 crimes were reported [1]. Also the rate of interpersonal violence in South Africa is the sixth highest in Africa and fifteenth in the world, with an intentional homicide rate of 31.8 per 100,000 population [2]. Research has shown that the determinants of crime and violence include poverty, inequality, racial tension and discrimination, psychological disorders and gender discrimination [3–7]. The consequences of crime and violence are far-reaching and extend from loss or damage to personal property to assault and death. While efforts have been put in place to reduce crime and violence in the country, such as the establishment of an inter-ministerial structure called the Justice Crime Prevention and Security Cluster (JCPS) whose chief objective is to reduce crime in the country, the problem remains widespread. If left unattended the consequences of crime of violence will affect the national economy and hopes for future development.

Youth, who are exposed to crime and violence in their families and communities, become victims and instigators. Research has shown that family violence has negative health and developmental consequences for youth including injuries, learning and concentration problems at school, and even death [8–10]. Equally worrisome is the finding that youth exposed to, and who are victims of crime and violence become perpetrators themselves [11]. This suggests a crime and violence cycle which perpetuates generation after generation. And in such circumstances, youth development is compromised which has greater implications on national growth and development.

Since 1994 the political environment in the country has been stable, with the African National Congress (ANC) remaining as the governing political body [12]. Despite the political stability however, national crime and violence rates were high at 38.6 murders per 100,000 population and 135.8 robberies per 100, 000 population from 2007 to 2008 [13]. Given that crime and violence is so widespread, it is often reported on in the television news, print media and more recently on social media. This increased awareness has made South Africans more cautious within their environments and has spread social mistrust within communities. It is therefore not only actual crime and violence, but the perceived safety within communities that threatens social cohesion and hampers efforts to reduce actual crime and violence in the country. Perceptions of crime create fear and panic, and this too affects the successful and health transition to adulthood for many youth.

An alternative exposure, which is beneficial to youth health and development, is participation in social activities. Sports, choirs, youth groups and other social activities are known to encourage physical exercise, team work skills, coordination, discipline and social communication skills [14, 15]. Outside of formal education, these activities are promoted as educational, healthy and vital for the development of youth. A recent study in South Africa found that encouraging young girls to take part in soccer, increased their confidence and HIV, Counselling and Treatment (HCT) uptake [16].

However, participation in social activities by youth in South Africa is hindered by cost, availability, access and competing interests such as part-time employment [17, 18]. Further, in a country where crime and violence is so high, youth in South Africa are limited in their freedom to participate in activities outside of their homes. While all of this is known, what remains to be determined is the relationship between perception of community safety and social activity participation behaviours among youth in South Africa. Perceived safety is a subjective measure which speaks to an individual’s state of mind including their fears and is partially based on their own experiences and that of others. As such, individuals who accept crime and violence as a norm are not likely to perceive their communities as risky, even if crime is high in these areas. For these individuals there is less fear, stigma and possibly more engagement in criminal activities. The purpose of this study is to identify the level of social activity participation among youth; determine the percentage of youth who perceive their communities as risky and test the association between youth social activity participation and perception of community safety in South Africa.

## Data

The data are from the nationally representative South African Youth Lifestyle Survey (SAYLS) 2008. The dataset is available by request from the Center for Justice and Crime Prevention (request website: http://www.cjcp.org.za/resources.html). Because this study was done using this secondary data, that has been completely de-personified, an ethics approval application was not needed. No persons were interviewed by any of the authors of the paper and we were not involved in data collection in any way. The authors of this manuscript did not have access to any identifying information about the respondents, such as their names, and we only analysed the completely anonymous data provided to us by the CJCP.

The sample design used is proportionately representative of the South African population. The sample frame was provided by Statistics South Africa 2001 Census data, and the sample was stratified by province and race [19]. A total of 550 enumerator areas (EA) were randomly selected and eight households in each EA was selected for the interview. Households were also randomly selected and where there were no youth (aged between 12 and 22 years old) in the selected household, or youth were not willing to participate in the survey, the next house on the list in the EA was selected [19]. In cases where the youth were below the age of 16 years, prior informed and written consent was obtained from primary caregivers or parents [19]. In addition, written consent was obtained from all youth participants in the survey. Final data was weighted by province, race and gender using the marginal totals drawn from the 2001 Census [19]. This was done to ensure accuracy of the representation of the experiences of young people in the country [19].

The interview used a questionnaire which included components exploring the respondents’ (a) demographics; (b) home environment; (c) feelings about and the nature of the community in which they live; (d) experiences at school; (e) exposure to violence; (f) sexual behaviours and so forth [19]. The CJCP weighted the data by province, race and sex using the marginal totals drawn from the 2001 Census [19]. This was done to ensure the most accurate representation of the experiences of young people throughout South Africa. A total of 4,391 youth aged 12–22 years old participated in the study [19]. The weighted total of youth is 10,502,705 representative of all 9 provinces and both sexes.

## Methods

### Study variables

Selected variables from the SAYLS on respondent’s demographic, socioeconomic, participation in social activities and perception of community safety characteristics were used [20]. Age, sex, race, highest level of education, type of place of residence, province of residence, number of household members, always enough to eat in the household, and positive view of the future were selected as control variables for the study.

Using Principle Component Analysis (PCA) a ‘perception of community safety’ index variable was created from eight variables on safety. PCA is performed on the data to create linear combinations from the set of variables and order the variables according to their contribution to the overall variability of the variables analysed. Based on the creation of this community safety index, risk quintiles are created which reflect the rankings of population by perception of community safety. Two quintiles were created within the index: risky and safe. The questions used in the creation of this variable are: (a) I would like to move out of my neighbourhood; (b) In my area there is lots of crime; (c) in my area there is lots of fights; (d) in my area it is easy to get a gun; (e) in my area it is easy to get a knife or other weapon; (f) I feel safe in my home; (g) I feel safe at school or work; (h) I fear travelling to or from school or work. The variable is categorised as ‘risky’ or ‘safe’. The five-point Likert scale responses were placed into quintiles in the PCA analysis. Responses that were ‘undecided’ were dropped from the analysis. Analysis of the Eigenvalues shows that 83% of the variance could be explained. The resulting variable has two categories which represent the components of the population that either feel their communities are risky (1) or safe (0), and this variable is used as the main predictor variable in the study.

The outcome of interest in this study is social activity participation. This study quantifies each type of social activity participation individually by sex of the youth and an index variable counting participation in at least one type of social activity was created. The questions from the survey used to determine this were: (a) Do you participate in youth/ religious groups? (b) Do you participate in sports? (c) Do you participate in any drama or theatre groups? (d) Do you participate in choir or singing groups?

### Statistical analysis

Weighted descriptive statistics of youth participation in social activities and participation by the main predictor and control variables in the study are shown. Chi-square tests were used to demonstrate the strength of the association between the outcome and independent variables in the study. Odds of social activity participation by perception of community risk is estimated by dividing the odds of participation among youth in risky communities, by the odds of participation among youth in safe communities [21]. Finally a binary logistic model was used, with participation in at least one social activity (yes/no) as the outcome variable [22]. This model was selected without an offset to examine the relationship between social activity participation, selected control variables and perception of community safety. The multivariate model produces odds ratios and variables found to be significant are considered as contributing factors to social activity participation. Statistical analysis was done using STATA 13 software.

## Results

Females take part in youth or religious groups (33.94%) the most. While half of the sample of male participants take part in sports. Overall, sports and youth or religious groups are the most popular social activities among youth, as seen in Table 1.

**Table 1:** Weighted distribution of social activity participation among youth in South Africa.

Social Activity	Males		Females		Total	
	Frequency	%	Frequency	%	Frequency	%
Youth/ Religious*	1,675,317	26.30	1,815,372	33.94	3,490,689	29.79
Sports Team*	3,238,494	50.84	1,722,809	32.21	4,961,303	42.34
Drama/ Theatre Group*	415,839	6.53	504,635	9.43	920,475	7.85
Choir/ Singing Group*	1,039,841	16.33	1,306,133	24.42	2,345,974	20.02
**Total**	**6,369,491**		**5,348,949**		11,718,440**	

*p-values <0.05;

** total is larger than sample because youth reported more than one social activity.

**Fig 1:**
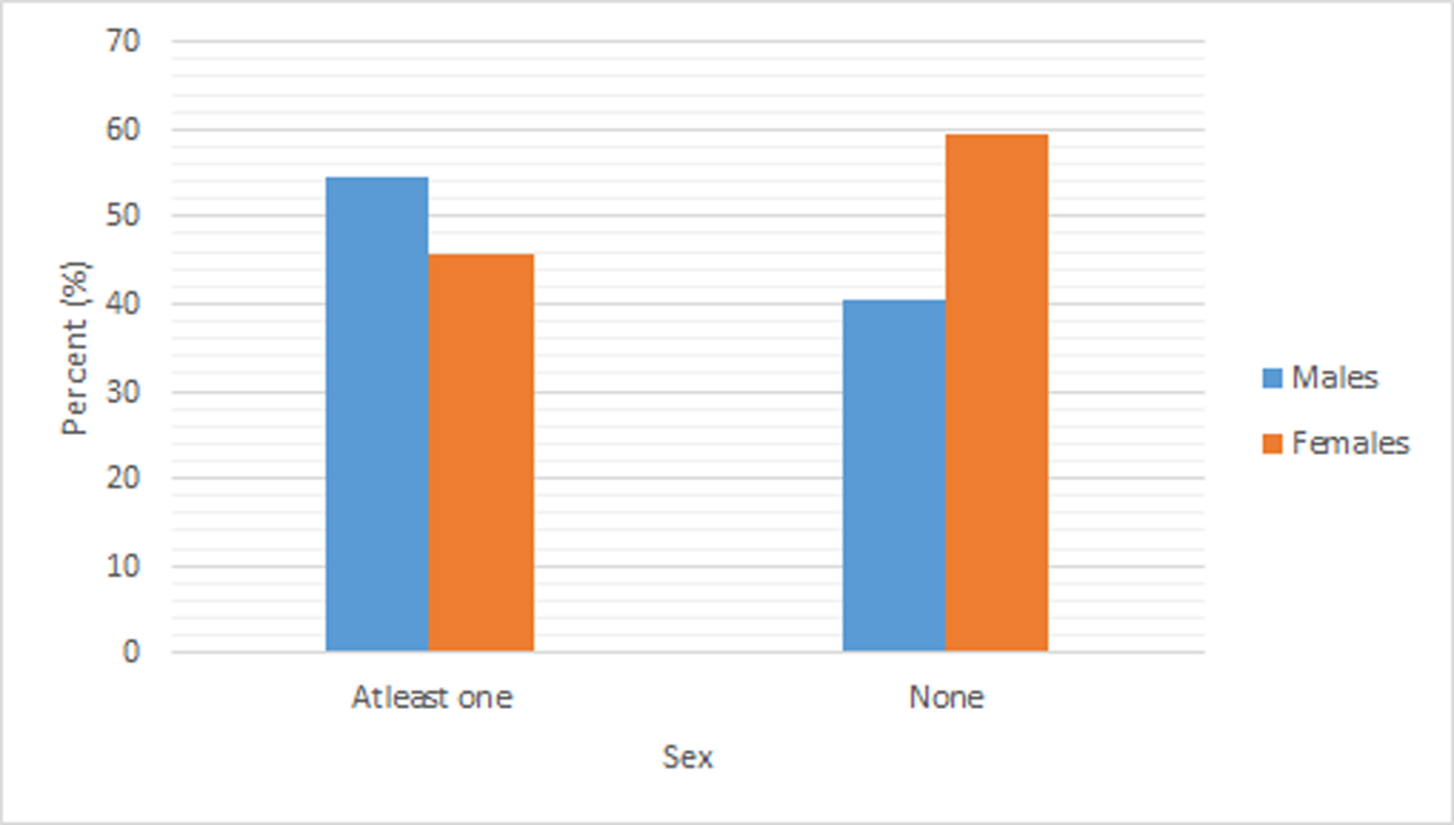
Weighted percentage distribution of social activity participation (at least one or none) by sex among youth in South Africa, 2008.

Males (54.46%) participated more in social activities than females (45.54%), as seen in Fig 1. There are more youth females who reported no social activities (59.49%) than males (40.54%).

**Fig 2:**
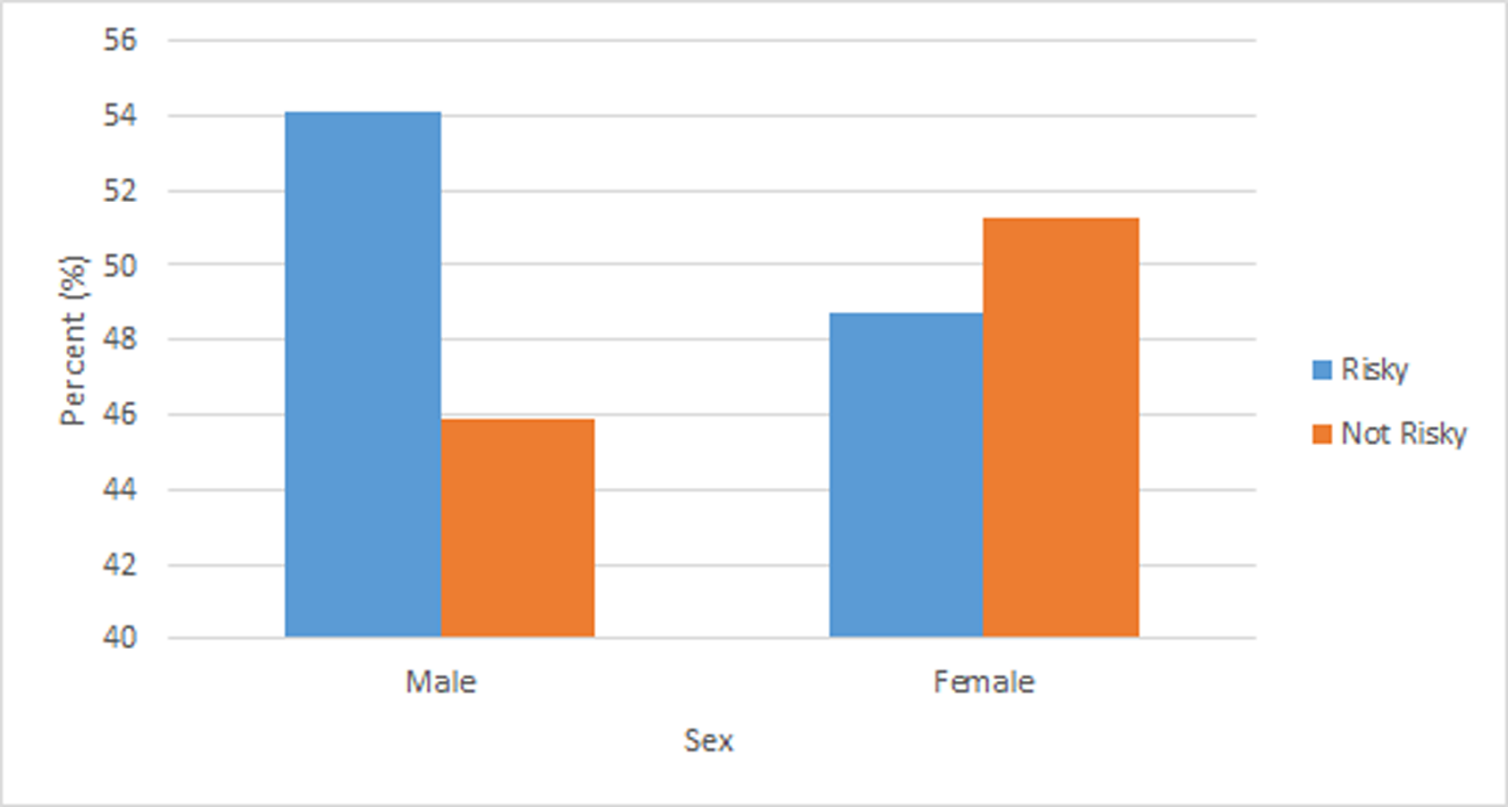
Weighted percentage distribution of percentage of community safety by sex among youth, South Africa, 2008.

Among males, 54.13% reported perceiving their community as risky, while 45.87% reported their communities as not risky (Fig 2). For females, 48.73% perceive their community as risky and 51.27% feel their communities are safe.

Among the sample of youth who participate in social activities in this study, 52.71% perceive their communities as risky (Table 2). By demographic characteristics of respondents who participate in atleast one social activity, 46.99% are between the ages of 15 and 19 years old; 84.14% are African/Black; 62.35% have between a Grade 9 and Grade 12 education; 53.64% live in households with five or fewer people; 77.52% reported their households having enough to eat; and 99.26% have an optimistic outlook of their future.

**Table 2:** Weighted characteristics of youth who participate in social activities in South Africa.

Characteristics	Social Activity Participation					
	At least one		None		Total	
Perception of community safety*	**Freq.**	**%**	**Freq.**	**%**	**Freq.**	**%**
Risky	3,780,512	52.19	1,755,896	53.87	5,536,408	52.71
Safe	3,462,791	47.81	1,503,507	46.13	4,966,297	47.29
Total**	7,243,303	100	3,259,402	100	10,502,705	100
Age groups*						
10–14	2,243,365	30.97	728,705	22.36	2,972,070	28.3
15–19	3,397,070	46.9	1,435,076	44.03	4,832,146	46.00
20–22	1,601,624	22.11	1,094,257	33.57	2,695,881	25.67
Unknown/ Missing	1,244	0.02	1,364	0.04	2,608	0.02
Total**	7,243,303	100	3,259,402	100	10,502,705	100
Sex*						
Male	3,944,421	54.46	1,321,343	40.54	5,265,764	50.14
Female	3,298,882	45.54	1,938,059	59.46	5,236,941	49.86
Total**	7,243,303	100	3,259,402	100	10,502,705	100
Race*						
White	496,216	6.85	157,586	4.83	653,802	6.23
African	6,094,563	84.14	2,701,673	82.89	8,796,236	83.75
Asian/indian	129,878	1.79	85,996	2.64	215,874	2.06
Colored	522,646	7.22	314,147	9.64	836,793	7.97
Total**	7,243,303	100	3,259,402	100	10,502,705	100
Highest level of education*						
Gr1- 8	2,650,762	36.6	1,006,841	30.89	3,657,602	34.83
Gr9-12	4,515,859	62.35	2,199,219	67.47	6,715,078	63.94
Unknown/ Missing	76,683	1.06	53,342	1.64	130,025	1.24
Total	7,243,303	100	3,259,402	100	10,502,705	100
Number of household members*						
< = 5	3,885,447	53.64	1,794,009	55.04	5,679,456	54.08
> = 6	3,357,857	46.36	1,465,393	44.96	4,823,250	45.92
Total**	7,243,303	100	3,259,402	100	10,502,705	100
Always enough to eat in the household*						
Yes	5,614,860	77.52	2,474,771	75.93	8,089,631	77.02
No	1,627,483	22.47	782,095	24	2,409,578	22.94
Unknown/ Missing	960	0.01	2,536	0.08	3,496	0.03
Total**	7,243,303	100	3,259,402	100	10,502,705	100
Positive view of the future*						
Yes	7,189,480	99.26	3,197,010	98.09	10,386,490	98.89
No	53,823	0.74	62,392	1.91	116,215	1.11
Total**	7,243,303	100	3,259,402	100	10,502,705	100

*p-values <0.05;

**Totals are less than Table 1 because here only participation in at least one social activity is measured, not multiple activities listed by some respondents

Table 3 shows the odds of social activity participation by characteristics of the respondents in the study. All variables were found to be statistically significant (p-values <0.05). Youth who perceive their communities as safe have only slightly higher odds (1.033) of participating in at least one social activity. Older youth (ages 15–22 years old) are less likely to participate in social activities compared to younger youth (RC = 1.00). Further, females (0.563); African (0.792); Asian/Indian (0.488) and Coloured (0.537) are also less likely to take part in social activities. Youth in higher grades (Gr9 to 12), living in households with more than 6 resident members and those without always enough to eat in the household were found to have increased odds of social activity participation. Youth with a negative view of the future are less likely to participate in social activities (0.441), compared to youth with a positive outlook of the future.

**Table 3:** Multivariate logistic regression showing the odds of social activity participation by characteristics of youth in South Africa, 2008.

Characteristics	Odds Ratio	[95% Conf.	Interval]
Perception of community safety*			
Risky	RC		
Safe	1.033	1.02977	1.03544
Age groups*			
`10–14	RC		
15–19	0.672	0.66909	0.67519
20–22	0.395	0.39272	0.39673
Sex*			
Male	RC		
Female	0.563	0.56174	0.56484
Race*			
White	RC		
African	0.792	0.78751	0.79734
Asian/indian	0.488	0.48291	0.49337
Colored	0.537	0.53334	0.54143
Highest level of education*			
Gr1- 8	RC		
Gr9-12	1.175	1.17006	1.17979
Number of household members*			
< = 5	RC		
> = 6	1.070	1.06709	1.073
Always enough to eat in the household*			
Yes	RC		
No	1.012	1.00818	1.01484
Positive view of the future*			
Yes	RC		
No	0.441	0.43595	0.44675

*p-values <0.05; RC denotes Reference Category

## Discussion

This study examines the associated likelihood of social activity participation and perceived community safety among youth in South Africa. The study has shown that the prevalence of participation in sports is higher among males than females. A study on gender differences in sport participation in the US found that teasing and body image concerns contribute to adolescent girls’ reduced rates of participation in sports and other physical activities compared to adolescent males [23]. A study on youth in South Africa also found that more males across all wealth quintiles participated in sport more than females [24]. This could be attributed to body image or popular sporting activities being delineated by sex, such as soccer and cricket for boys and netball for girls, and that male sports receive more public attention than female sports.

Participation in at least one type of social activity was given consideration in this study and it was found that more males participate in at least one type of social activity than females. In African societies, adolescent females assume many roles. From a young age, females are trained to do household chores such as cooking, cleaning and are even caregivers to younger siblings [25, 26]. Given these expectations, coupled with attending school and doing homework, there is limited time for young females to engage in several social activities. While African male youth do not have the same roles and responsibilities, their time is free to engage in sports and additional extracurricular activities.

Perception of community safety is the main predictor variable of this study and it was found that more than half the males of the study perceived their communities as risky, while less than half of the females felt the same. Males are often more affected and involved in crime and violence than females. Studies have shown that the rate of male homicides is higher at 6 male for every 1 female victim in South Africa [27]. Further, the estimated number of males in prison far outweighs the number of females at 156,000 males to 4,000 females in the country [28]. Given their greater involvement, it therefore stands to reason then that males are more aware of criminal activities in their surroundings than females. This awareness could be because males are either directly or indirectly (through knowledge of their friends and neighbours) involved in criminal activities in their communities.

Related to this, among all youth who participate in at least one social activity, more than half perceive their communities as risky. Similarly among all youth who do not participate in at least one social activity, more than half perceive their community as risky. This reflects national crime statistics which show a high prevalence of crime and violence in the country [1]. This is also aligned with results from a recent national survey which found that 41.3% of South Africans felt that crime had increased in the country from 2010 to 2013 [29]. Further, it was found that the crimes most feared by South African’s are housebreaking or burglary (59.7%); sexual assault (30.5%) and physical assault (25.5%) [29]. Also 45.1% of South Africans felt it is very unsafe to walk around their communities at night [29].

Males are more likely to participate in social activities if they perceive their communities as risky. There are two suggestable hypotheses for this finding. First, is that social activity participation provides a safe environment for youth who want to escape the crime and violence which surrounds their living conditions. As such, youth either electively choose extracurricular social activities or are encouraged by parents and peers to join so as to avoid engaging in deviant behaviour. This hypothesis is supported by literature that has found the protective effect of social activities from harmful behaviours [30, 31]. One study found that youth engagement in social activities reduced depression symptoms and suicidal ideation [32]. Though, a sex differential has been noted with one study finding that involvement in extracurricular activities including sport, had a protective effect from alcohol use among young males in Poland, but no effect on females [33]. These results are supportive of the Social Control Theory which posits that youth who have stronger attachment and involvement in positive social norms and activities are less likely to engage in deviant behaviour [34].

However, there is a second possible explanation for why youth, particularly males, are more likely to engage in social activities if they perceive their communities as risky. This could be because they are using social activities to engage in risky and deviant behaviours with peers. A study from the UK found that 89% of under-aged youth aged 15 and 16 years old who were members of youth clubs, groups or teams consumed alcohol [35]. Another study found that sports participation increased aggressive behaviour among youth in Scotland [36]. And while it is acknowledged that the role of individual motivation in sports participation, suggesting that it is possible that physically aggressive youth choose sports over other social activities, the authors also acknowledge that the supervision and organisation of the sporting activities contribute to whether this is a risk or protective factor for youth [36]. These results are supportive of Differential Association Theory which posits that youth engagement in criminal and deviant behaviour is because of the environment and communities they are exposed to, where such behaviours are deemed acceptable and held in a positive regard [37, 38]. However moot the literature is on the role of social activities on risky behaviours, what is clear is that it cannot be assumed that extracurricular sports, clubs and groups have only a positive relationship with youth behaviour outcomes.

## Conclusion

There exists an association between social activity participation and perception of community safety. However, the statistical difference is weak and therefore there is need for further research in this area. The benefits of social activity participation to youth development are known. However, the rationale for involvement and its stance as a protective factor against crime and violence is unclear. A research done on Swedish adolescents found that seeing friends, doing something fun or interesting were the main motivations for social activity participation [39]. However, this type of qualitative inquiry is missing in the South African context where motivations may differ based on socio-cultural and crime differences in the two countries. In addition, research on the behaviours of youth at social activities is needed to determine if this is in fact a risk or protective factor against community crime and violence.

### Limitations of the study

Due to the cross-sectional nature of the data, temporal sequencing cannot be established. That is, the study is unable to determine whether perception of community safety is the cause of social activity participation among participants. Also the large sample of youth in the study affects the statistical significance of the results in favour of significance. Further there is no guarantee that respondents honestly and accurately reported on personal, sensitive and traumatising events such as domestic violence. However, interviewers were instructed to re-emphasise the confidentiality and anonymity of all data collected and also gave respondents the opportunity to refuse to answer any questions they were uncomfortable answering [19]. Further, there are youth who did not participate in the survey who may have been more affected by community violence. This means that there is the possibility that community violence and social activity participation is underestimated in this study.
